# Genome sequence analysis of a Helicoverpa armigera single nucleopolyhedrovirus (HearNPV-TR) isolated from *Heliothis peltigera* in Turkey

**DOI:** 10.1371/journal.pone.0234635

**Published:** 2020-06-12

**Authors:** Gozde Busra Eroglu, Cihan Inan, Remziye Nalcacioglu, Zihni Demirbag

**Affiliations:** 1 Department of Biology, Faculty of Science, Karadeniz Technical University, Trabzon, Turkey; 2 Department of Molecular Biology and Genetics, Faculty of Science, Karadeniz Technical University, Trabzon, Turkey; The University of Queensland, AUSTRALIA

## Abstract

The entire genome of Helicoverpa armigera single nucleopolyhedrovirus (HearNPV-TR) was sequenced, and compared to genomes of other existing isolates. HearNPV-TR genome is 130.691 base pairs with a 38.9% G+C content and has 137 open reading frames (ORFs) of ≥ 150 nucleotides. Five homologous repeated sequences (*hrs*) and two baculovirus repeated ORFs (*bro*-a and *bro*-b) were identified. Phylogenetic analysis showed that HearNPV-TR is closer to HaSNPV-C1, HaSNPV-G4, HaSNPV-AU and HasNPV. However, there are significant differences in *hr*3, *hr*5 regions and in *bro*-a gene. Pairwise Kimura-2 parameter analysis of 38 core genes sequences of HearNPV-TR and other Helicoverpa NPVs showed that the genetic distances for these sequences were below 0.015 substitutions/site. Genomic differences as revealed by restriction profiles indicated that *hr3*, *hr5* regions and *bro*-a gene may play a role in the virulence of HearNPV-TR.

## Introduction

The *Baculoviridae* is a family of rod shaped, enveloped virions with circular, double-stranded DNA genomes [1]. Members of this family infect insect in the orders of Lepidoptera, Hymenoptera and Diptera. These viruses have been known since 1911, and some members were successfully developed as biocontrol agent against agricultural and forest insect pests. Baculoviruses have also been developed as vectors for the expression of exogenous genes employed in a variety of sectors such as the pharmaceutical, medical and biotechnological [2, 3]. *Baculoviridae* is divided into 4 genera, *Alphabaculovirus* (lepidopteran Nucleopolyhedroviruses), *Betabaculovirus* (lepidopteran Granuloviruses), *Gammabaculovirus* (hymenopteran NPVs) and *Deltabaculovirus* (dipteran NPVs) [4]. Alphabaculoviruses are further subdivided into two groups, I and II. Group I alphabaculoviruses contain GP64 as the envelope fusion protein in the budded virus phenotype whereas group II alphabaculoviruses as well as betabaculoviruses and deltabaculoviruses contain F as the membrane fusion protein [5–7].

Having the total genome sequence of a baculovirus is useful in designing intelligent strategies for biotechnological use of the virus including pest control [8]. To date, the total sequences of 79 baculovirus genome have been deposited in the National Center for Biotechnology Information (NCBI, www.ncbi.nlm.nih.gov) of which, 51 belong to *Alphabaculovirus*, 24 to *Betabaculovirus*, 1 to *Deltabaculovirus* and 3 to *Gammabaculovirus*. The genome of AcMNPV, which is the architype lepidopteran NPV, was sequenced in 1994 [9].

*Helicoverpa armigera* (Hubner) (Lepidoptera: Noctuidae), a polyphagous insect is recognized as one of the most economically important pests of agriculture worldwide. If this species, which is active during summer, is not controlled, it will cause serious damage to many agricultural products [10, 11]. We have previously isolated a baculovirus from *Heliothis peltigera* larvae collected from a safflower field in Adana of southern Turkey. However, Kimura analysis showed that this virus is actually a strain of H. armigera single nucleopolyhedrovirus, which belongs to the group II *Alphabaculoviruses* [11]. Therefore, the isolate was named as HearNPV-TR. Interestingly, this isolate has significantly high virulence to four Heliothine species (*H*. *armigera*, *H*. *peltigera*, *Heliothis viriplaca* and *Heliothis nubigera*) distributed in Turkey [12].

In the current study, we sequenced and analyzed the whole genome of HearNPV-TR and compared it to the other fully sequenced Helicoverpa NPV genomes deposited in the NCBI database. The HearNPV-TR genome was found to have 130.691 base pair (bp) genome size.

## Materials and methods

### Virus propagation and occlusion body (OB) purification

The HearNPV-TR was propagated by the droplet feeding method in third instar larvae of *H*. *armigera* [13]. The droplet feeding solution (2% red food dye and 20% feeding stimulant: sucrose) and virus suspension at 2 x 10^7^ OB/ml were mixed in a 1:1 ratio. One microliter of this mixture was given to each of the 100 larvae that were fasted for 24 hours. Because of the cannibalistic characteristics of *H*. *armigera* after the third larval instar, larvae were reared individually in plastic boxes containing blocks of artificial diet [14] and incubated at 26°C and 60% humidity. Larvae exhibiting symptoms of infection were collected daily and stored at + 4°C. Larvae were homogenized in water (200 μl dH_2_O per larvae) and filtered through a double layer of cheesecloth to remove debris. OBs were purified according to the procedure described by Munoz et al. [15] and counted in a haemocytometer.

### Viral DNA extraction

OBs (10^9^ OB/ml) were dissolved by mixing with 3X DAS buffer (0.3 M Na_2_CO_3_, 0.5 M NaCl, 0.03 M EDTA; pH 10.5) at 37°C for 30 min. Non-dissolved OBs were removed by centrifugation at 1000 rpm for 5 min. The supernatant fluid containing occlusion derived virus (ODV) was centrifuged at 20,300×g for 30 min. DNA was extracted from virus particles according to Reed et al. [16] and dialyzed for 24 hours against 4 changes of 0.1X TE buffer (1 mM Tris-HCl, 0.1 mM EDTA, pH 8.9). DNA concentration and purity were determined by spectrophotometry and electrophoresis.

### Next generation sequencing and genome assembly

HearNPV-TR full genome was sequenced and assembled by Macrogen Inc., Soul Korea. The RSII Pacific Biosciences (PacBio) single molecule platform was used for genome sequencing. PacBio SMRTbell(TM) system performs multiple sequencing of generated circular template and thus, comes with high accuracy. Hairpin adapter ligated to both ends of the dsDNA to make a single-stranded circular template called SMRTbell. Then all templates were loaded to SMRT cells including fluorescent bound dNTPs for real time sequencing by a polymerase. While polymerase add a new base system generates a movie of light pulses. Then the pulses were converted into sequence files. After the sequencing process, the first step is *de novo assembly* to build contigs of the genome using generated sequence files. Firstly, a preassembly step was performed, which consisted of a map representing the raw state of long and single pass read. From the mapped reads, a long high accuracy consensus sequence of the target genome was obtained. *De novo* assembly analysis performed with Unicycler software (v0.4.6) with normal mode and minimum fasta length 100 options. For this the reads that fully contained in other reads and reads that had too high or too low overlaps were filtered out. Subsequently, HiSeq reads were applied for sequence compensation to construct contigs more accurately. Thereafter, whole genome was assembled and the locations of protein-coding sequences were identified. The total numbers of GC (%) and quality scores [Q20 (%), and Q30 (%)] were detected. The crude reads were 13,084,430, GC content was 39.26%, and Q30 was 86.13%. Contigs with overlapping end were connected to form a circular contig. The next step was correcting and filtering the reads using HiSeq reads to generate accurate genome sequence. This error correction analysis performed with Pilon software (v1.21). After all, full genome of HearNPV-TR was created as one contig and continued with annotation process using online Benchling Biology Software (Retrieved from https://benchling.com).

### Genome annotation, phylogeny and Kimura-2 parameter analysis

The reference HaSNPV-AU genome (Accession number: KJ909666) was used to determine the location of all ORFs and *hrs* in the genome. All ORFs potentially encoding proteins with 50 or more amino acids were detected and annotated in the Benchling Biology Software (retrieved from https://benchling.com). Concatenated amino acid sequences encoded by the 38 conserved core genes belonging to 51 baculovirus genomes and HearNPV-TR genome were aligned using the BioEdit (7.1.3.0) program. In phylogenetic analysis, the Jones-Taylor-Thornton (JTT) model with 1000 bootstrap in the Maximum Likelihood method to generate a phylogeny, the 38 core protein sequences were used by MEGA6 program. To determine the ratio of transitions to transversions, we have used Kimura-2 parameter analysis. This analysis are often used to analyze phylogenetic similarity/distance between baculovirus species. These distances were estimated from the alignment of all 38 core gene sequence of the 52 baculovirus species and aligned in the BioEdit program, and the Kimura analysis was performed by MEGA6 software.

### Comparison of restriction endonuclease profiles

Two micrograms of HearNPV-TR DNA were separately digested with XhoI and KpnI restriction endonucleases (Biolabs) at 37°C for 4 hours. Also, lambda DNA digested with HindIII and NarI restriction enzymes were used as marker. The samples were electrophoresed on a 0.6% agarose gel at 16 Volt overnight. The restriction profile of the HearNPV-TR genome was compared to those of the genomes of HaSNPV (MG569706), HaSNPV-C1 (AF303045), HaSNPV-G4 (AF271059) and HaSNPV-AU (JN584482) by using the Benchling Biology Software (Retrieved from https://benchling.com).

## Results

### Genome properties of HearNPV-TR

**Fig 1:**
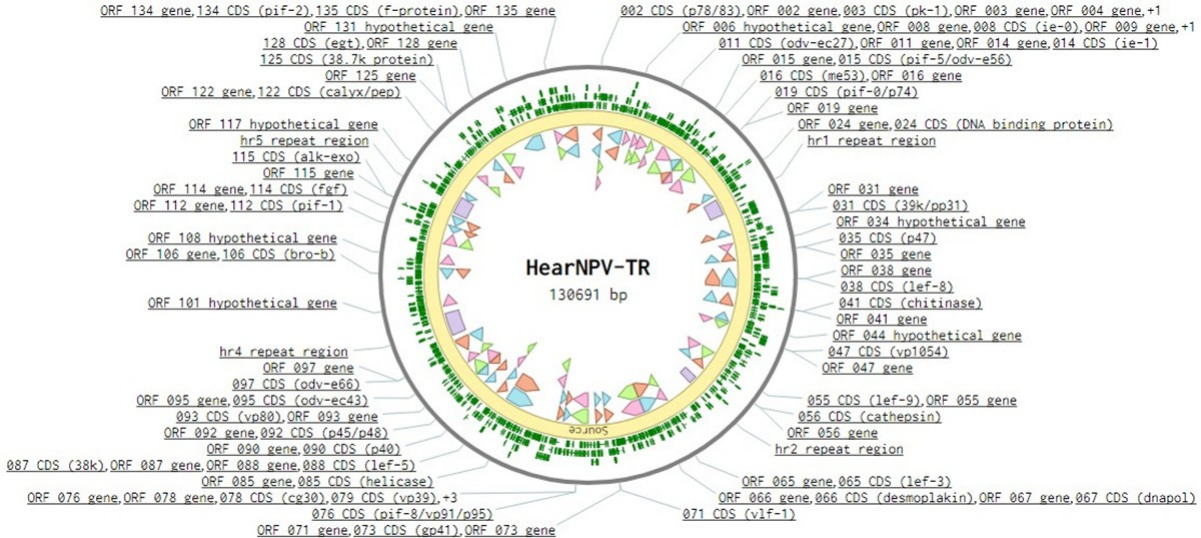
Circular genome map of sequenced HearNPV-TR genome.

The full genome of the HeaNPV-TR isolate was analyzed and deposited at NCBI (Accession number: MK507817). The genome size of HearNPV-TR was determined to be 130.691 kb in length with 38.9% GC content. The locations and directions of all ORFs in the genome are shown in the circular map in Fig 1 with the first nucleotide of the ATG start codon of the polyhedrin gene being number one of the sequence [17–19]. Sixty-nine ORFs are located in a clockwise direction and 68 in a counterclockwise direction with respect to the transcriptional orientation of the polyhedrin gene. HearNPV-TR ORFs were compared to 6 homologues in other baculoviruses; Autographa californica MNPV (AcMNPV, *Alphabaculovirus* I), Cydia pomonella GV (CpGV, *Betabaculovirus*) and, four Helicoverpa SNPVs (H. assulta NPV, H. armigera C1, H. armigera G4, and H. armigera AU).

The HearNPV-TR genome contains 137 ORFs, 39 of which are of unknown function and, hence, were annotated as encoding hypothetical proteins. All these hypothetical genes have homologues in other Helicoverpa SNPV genomes. The annotated ORFs include the 38 core genes of family *Baculoviridae*. While HearNPV-TR genome shared 98% nucleotide sequence identity with HaSNPV and HaSNPV-C1 genomes, it showed 99% nucleotide identity with HaSNPV-G4 and HaSNPV-AU genomes.

### ORFs functions

As in other baculoviruses, the genome of HearNPV-TR contains structural genes, auxiliary genes, genes related to replication, transcription, oral infectivity and genes of unknown function (Table 1). Nine genes are related to DNA replication, four of which are core genes: *dnapol* (ORF67), *helicase* (ORF85), *lef*-2 (ORF119) and *lef*-1 (ORF126). The remaining five (*ie*-1/ORF14; *me*53/ORF16; *dbp*/ORF24; *lef*-11/ORF32; *lef*-3/ORF65) are lepidopteran baculovirus conserved genes. Other DNA replication genes not found in the HearNPV-TR genome are *helicase*-2, *dna-ligase*, *dUTPase*, *RNase reductase*-1, *RNase reductase*-2, *pcna*, *lef*-7, *ie*-2, *pe*38 [20].

**Table 1:** Classification of functional genes in HearNPV-TR genome.

**Replication**	*ie-1* (ORF14), *me53* (ORF16), *dbp* (ORF24), *lef-11* (ORF32) *lef-3* (ORF65), ***dnapol*** (ORF67), ***helicase*** (ORF85), ***lef-2*** (ORF119), ***lef-1*** (ORF126)	*helicase-2*, *dna-ligase*, *dUTPase*, *RNase reductase-1*, *RNase reductase-2*, *pcna*, *lef-7*, *ie-2*, *pe38*
**Transcription**	*ie-0* (ORF8), *lef-6* (ORF23), *39k/pp31* (ORF31), ***p47*** (ORF35), *lef-12* (ORF36), ***lef-8*** (ORF38), *lef-10* (ORF46), ***lef-9*** (ORF55), *he65* (ORF61), *putative met transf*. (ORF63), ***vlf-1*** (ORF71), ***lef-4*** (ORF80), ***lef-5*** (ORF88), *pkip-1* (ORF132)	
**Structural proteins (**Packaging, assembly, and release)	*polh* (ORF1), *p78/83* (ORF2), *pk-1* (ORF3), ***odv-e18*** (ORF10), ***odv-ec27*** (ORF11), *p10* (ORF20), ***vp1054*** (ORF47), *fp* (ORF53), *gp37* (ORF58), ***gp41*** (ORF73), ***vp39*** (ORF79), ***odv-e25*** (ORF83), ***p6*.*9*** (ORF89), *odv-e66* (ORF97), *p24* (ORF120), *gp16* (ORF121), *calyx* (ORF122), *odv-c21* (ORF124), *f protein* (ORF135)	*vef-1*, *vef-2*, *vef-3*, *gp50*, *gp64*
**Auxiliary (**Cell cycle arrest and/or interaction with host proteins)	*ubiquitin* (ORF28), *chitinase* (ORF41), *cathepsin* (ORF56), *sod* (ORF107), *fgf* (ORF114), ***alk-exo*** (ORF115), *egt* (ORF128), *arif-1* (ORF133)	*ptp-1*, *ptp-2*, *ctl-1*, *ctl-2*
**Oral infectivity**	***pif-5/odv-e56*** (ORF15), ***pif-0/p74*** (ORF19), ***pif-6*** (ORF64), ***pif-8/vp91/p95*** (ORF76), ***pif-4*** (ORF86), ***pif-7*** (ORF94), ***pif-9/ac108*** (ORF96), ***pif-3*** (ORF99), ***pif-1*** (ORF112), ***pif-2*** (ORF134)	
**Apoptosis-inhibiting proteins**	*iap-2* (ORF62), *iap-3* (ORF104)	*iap-1*, *iap-4*, *p35/ac135*
**Unknown**	***p49*** (ORF9), *ac145* (ORF12), *ac146* (ORF13), *ac26* (ORF25), *lese25* (ORF30), *ac38* (ORF33), *ac43* (ORF37), *ac52* (ORF42), ***ac53*** (ORF43), *ac56* (ORF49), *ac57* (ORF50), *ac59* (ORF51), *chaB* (ORF52), ***desmoplakin*** (ORF66), *ac74* (ORF68), *ac76* (ORF70), ***ac78*** (ORF72), ***ac81*** (ORF74), *telokin* (ORF75), ***p33*** (ORF81), ***p18*** (ORF82), ***38k*** (ORF87), ***p40*** (ORF90), ***p48/p45*** (ORF92), ***odv-ec43*** (ORF95), *p13* (ORF98), *ac106* (ORF102), *ac117* (ORF111), *ac111* (ORF118), *38*.*7k protein* (ORF125)	*ac4*, *ac5*, *ac7*, *ac11*, *ac12*, *ac13*, *ac17*, *ac18*, *ac19*, *ac29*, *ac30*, *ac33*, *ac34*, *ac39*, *ac44*, *ac45*, *ac55*, *ac58*, *ac63*, *ac70*, *ac72*, *ac73*, *ac75*, *ac84*, *ac85*, *ac87*, *ac91*, *ac97*, *ac107*, *ac110*, *ac112*, *ac113*, *ac114*, *ac116*, *ac118*, *ac120*, *ac121*, *ac122*, *ac124*, *ac132*, *ac140*, *ac149*, *ac150*, *ac152*, *ac154*
**Others**	hypothetical (ORF5, ORF6, ORF7, ORF17, ORF18, ORF22, ORF26, ORF27, ORF29, ORF34, ORF39, ORF40, ORF44, ORF45, ORF48, ORF54, ORF57, ORF60, ORF69, ORF77, ORF84, ORF91, ORF100, ORF101, ORF103, ORF105, ORF108, ORF109, ORF110, ORF113, ORF116, ORF117, ORF123, ORF127, ORF129, ORF130, ORF131, ORF136, ORF137), *hoar* (ORF4), *p26* (ORF21), *vp80* (ORF93), *bro-a* (ORF59), *cg30* (ORF78), *bro-b* (ORF106)	
**Oral infectivity**	***pif-5/odv-e56*** (ORF15), ***pif-0/p74*** (ORF19), ***pif-6*** (ORF64), ***pif-8/vp91/p95*** (ORF76), ***pif-4*** (ORF86), ***pif-7*** (ORF94), ***pif-9/ac108*** (ORF96), ***pif-3*** (ORF99), ***pif-1*** (ORF112), ***pif-2*** (ORF134)	
**Apoptosis-inhibiting proteins**	*iap-2* (ORF62), *iap-3* (ORF104)	*iap-1*, *iap-4*, *p35/ac135*
**Unknown**	***p49*** (ORF9), *ac145* (ORF12), *ac146* (ORF13), *ac26* (ORF25), *lese25* (ORF30), *ac38* (ORF33), *ac43* (ORF37), *ac52* (ORF42), ***ac53*** (ORF43), *ac56* (ORF49), *ac57* (ORF50), *ac59* (ORF51), *chaB* (ORF52), ***desmoplakin*** (ORF66), *ac74* (ORF68), *ac76* (ORF70), ***ac78*** (ORF72), ***ac81*** (ORF74), *telokin* (ORF75), ***p33*** (ORF81), ***p18*** (ORF82), ***38k*** (ORF87), ***p40*** (ORF90), ***p48/p45*** (ORF92), ***odv-ec43*** (ORF95), *p13* (ORF98), *ac106* (ORF102), *ac117* (ORF111), *ac111* (ORF118), *38*.*7k protein* (ORF125)	*ac4*, *ac5*, *ac7*, *ac11*, *ac12*, *ac13*, *ac17*, *ac18*, *ac19*, *ac29*, *ac30*, *ac33*, *ac34*, *ac39*, *ac44*, *ac45*, *ac55*, *ac58*, *ac63*, *ac70*, *ac72*, *ac73*, *ac75*, *ac84*, *ac85*, *ac87*, *ac91*, *ac97*, *ac107*, *ac110*, *ac112*, *ac113*, *ac114*, *ac116*, *ac118*, *ac120*, *ac121*, *ac122*, *ac124*, *ac132*, *ac140*, *ac149*, *ac150*, *ac152*, *ac154*

All baculovirus transcription genes are present in HearNPV-TR genome, six of which (*p*47/ORF35, *lef*-8/ORF38, *lef*-9/ORF55, *vlf*-1/ ORF71, *lef*-4/ORF80, *lef*-5/ORF88) are core genes.

There are total of nineteen structural genes in the HearNPV-TR genome responsible for packaging, assembly, and release. On the other hand, *vef*-1, *vef*-2, *vef*-3, *gp*50 and *gp*64, also known as structural genes, are not detected in the genome.

Eight auxiliary genes related to cell cycle arrest and interaction with host proteins are found in HearNPV-TR genome. The *ptp*-1, *ptp*-2, *ctl*-1 and *ctl*-2, also known as auxiliary genes, are not found in the genome.

To date, all baculoviruses encode ten proteins essential for oral infection and are known as *per os* infectivity factors (PIFs). All ten baculovirus *pif* genes, *pif*-0—*pif*-9 have been identified in the HearNPV-TR genome.

Baculoviruses generally contain two types of anti-apoptotic genes; *p35* and inhibitors of apoptosis (*iap*) [21]. However, HearNPV genome does not contain *p35*, and contains two *iap* genes (*iap*-2/ORF62; *iap*-3/ORF104).

**Fig 2:**
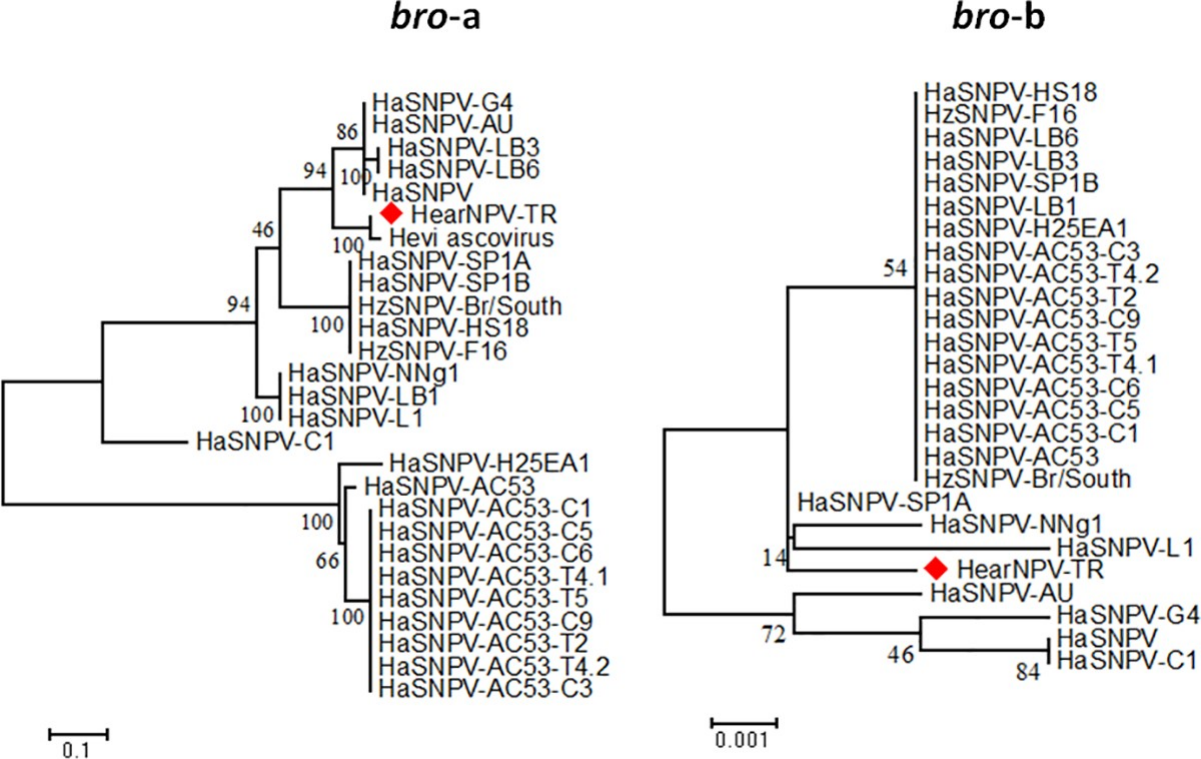
Phylogenetic analysis of *bro*s genes.

Baculovirus repeated ORFs (*bro*s) are present in some invertebrate DNA viruses [22]. Two *bro* genes, *bro*-a and *bro*-b, encoding 142 and 502 amino acid respectively, were identified in the HearNPV-TR genome. According to Blast analysis, *bro*-a gene of HearNPV-TR presents high identity to *bro* 23 gene of Heliothis virescens ascovirus 3i isolate (HvAV-3i; accession no: AXN77341). A phylogenetic analysis performed using the *bro*-a genes of all HearNPV species and *bro*-23 gene of HvAV-3i supported this relation (Fig 2). However, *bro*-b of HearNPV-TR has 99% identity to those of the other HearNPV species (S1 Table). Phylogenetic analysis also supported the difference of *bro*-a and the identity of *bro*-b genes (Fig 2) of HearNPV-TR to those of other HearNPV species.

Homologous repeat sequences (*hrs*) are commonly found in baculovirus genomes. These repeats act as enhancers of gene expression and may act as origins of DNA replication [23–28]. Five *hrs* recognized in the HearNPV-TR genome ranged from 714 bp to 3246 bp in size (S1 Table).

### Phylogeny and Kimura-2 parameter analysis

**Fig 3:**
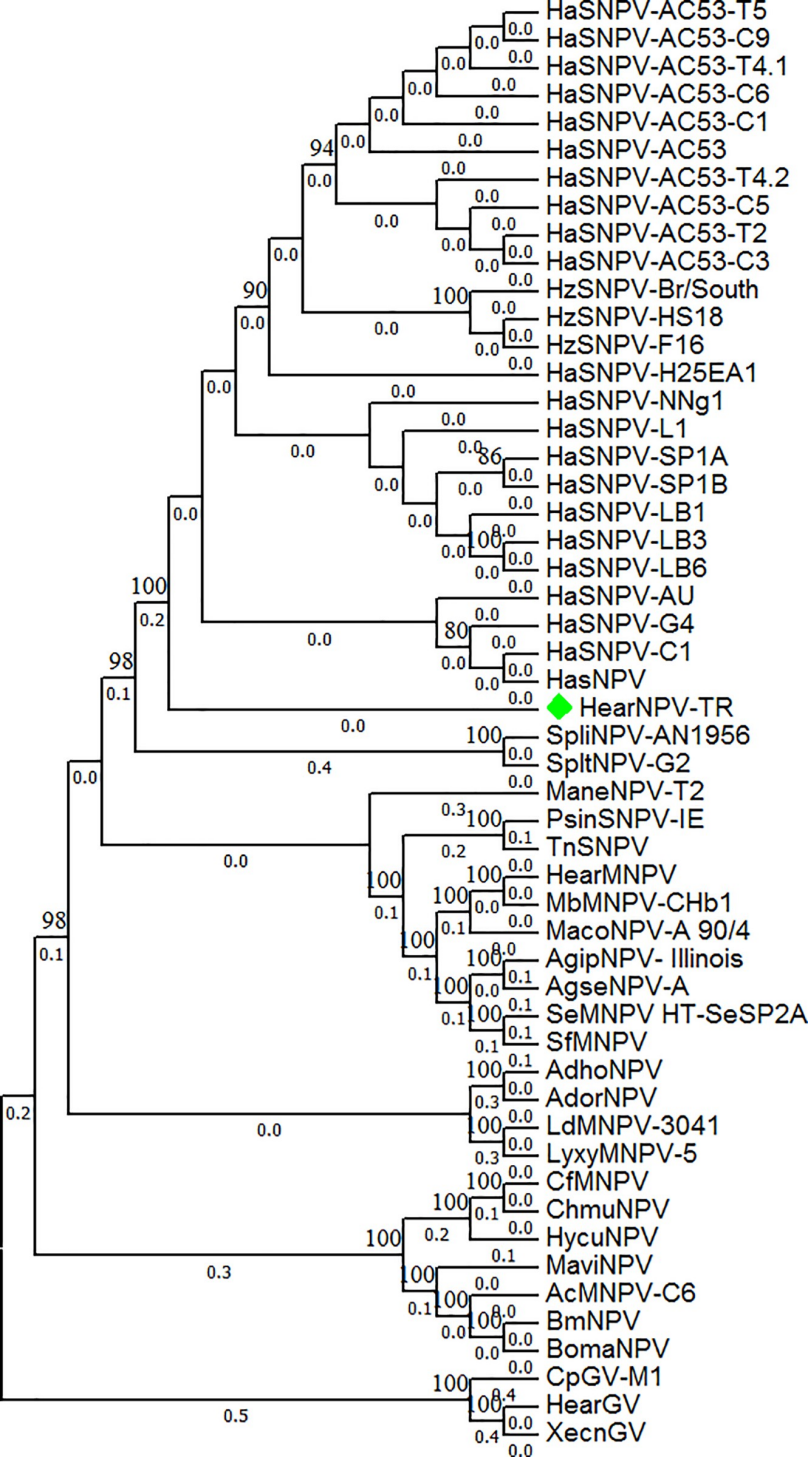
Phylogenetic analysis of HearNPV-TR based on concatenated sequences encoded by all 38 baculovirus core genes.

The amino acid sequences encoded by the 38 core genes from 51 baculoviruses in the genera *Alphabaculovirus* and *Betabaculovirus* were used to construct phylogenetic trees. Detailed information on the baculovirus genomes used in phylogenetic trees is shown in S2 Table. The genome of Helicoverpa armigera NPV-TR (HearNPV-TR) clustered close to the Helicoverpa armigera China (-C1 and -G4), Helicoverpa armigera Australia, and Helicoverpa assulta China NPV strains (Fig 3).

Kimura-2 parameter analysis was performed to define baculovirus species based on nucleotide sequence distances [29]. This analysis was used to address the position of HearNPV-TR among the Helicoverpa NPVs. Two baculoviruses are considered the same species if the nucleotide locus distances value is less than 0.015 [29]. To date, all Helicoverpa NPV isolates have been accepted as variants of HearNPV [30]. Based on concatenated amino acid sequences of the 38 core genes, the nucleotide locus distances between Helicoverpa NPV isolates and HearNPV-TR was found to be less than 0.015, which revealed that HearNPV-TR is a variant of the H. armigera SNPV (S3 Table).

### Comparison of restriction endonuclease profiles

**Fig 4:**
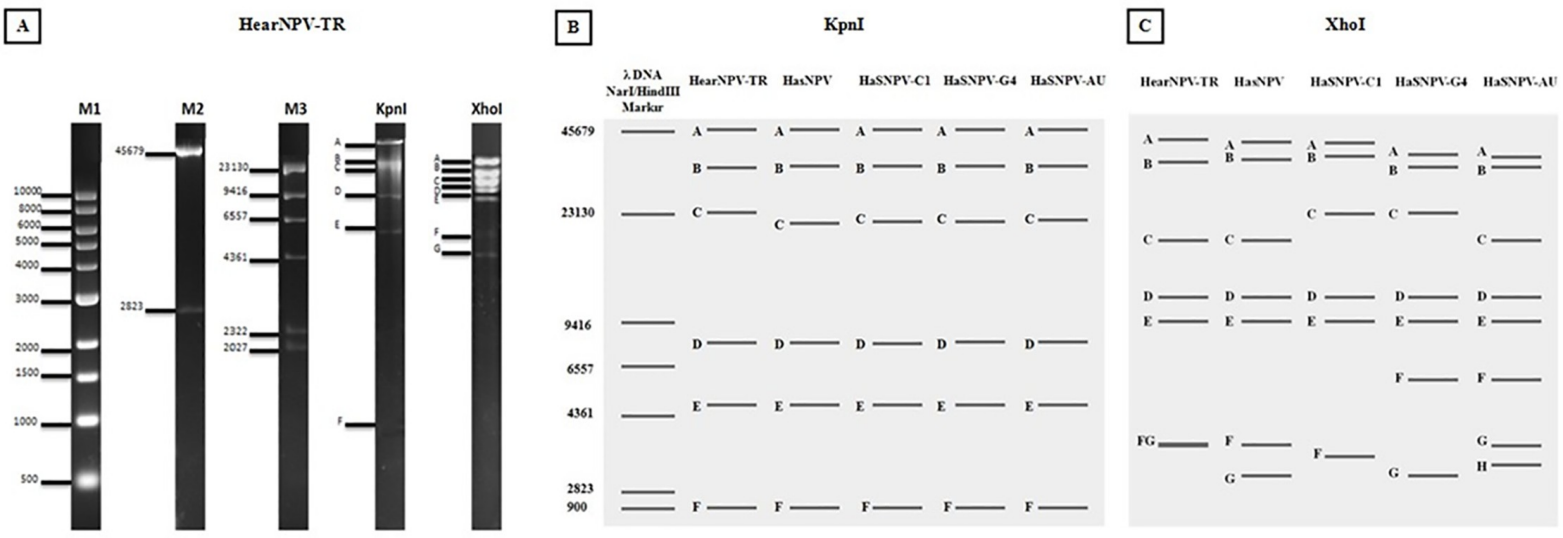
Restriction endonuclease (KpnI and XhoI) profiles. A: Restriction analysis by electrophoresis of HearNPV-TR, B-C: Restriction endonuclease profiles (KpnI and XhoI) of HearNPV-TR, HasNPV, HaSNPV-C1, -G4, -AU based on the full genome sequence using Benchling online tool.

Digestion of the HearNPV-TR genome with KpnI and XhoI restriction endonucleases generated 6 and 7 fragments, respectively (Fig 4A). The genomes of HearNPV-TR, HaSNPV, HaSNPV-C1, HaSNPV-G4 and HaSNPV-AU were digested with KpnI and XhoI *in silico* by using the Benchling program and compared to HearNPV-TR (Fig 4B and 4C, Table 2). The restriction profiles showed that HearNPV-TR has differences from those of HaSNPV, HaSNPV-C1, HaSNPV-G4 and HaSNPV-AU. Differences in restriction endonuclease profiles are normally observed among different geographical isolates and are a consequence of alterations in restriction sites, insertions, deletions and presence or absence of tandemly repeated sequences [31–33].

**Table 2:** Restriction endonuclease profile of HearNPV-TR, HasNPV, C1, G4, AU strains as per KpnI and XhoI enzymes.

Fragment	KpnI					XhoI				
	HearNPV-TR	HasNPV	HaSNPV-C1	HaSNPV-G4	HaSNPV-AU	HearNPV-TR	HasNPV	HaSNPV-C1	HaSNPV-G4	HaSNPV-AU
A	**54.8**	55.5	56.3	56.8	56.0	**41.8**	41.2	40.8	37.5	36.7
B	**34.5**	34.9	34.9	34.9	34.9	**35.6**	36.2	37.0	34.2	34.3
C	**24.8**	22.8	23.1	23.1	23.4	**19.9**	20.0	24.4	24.5	20.0
D	9.5	9.5	9.4	9.5	9.5	**13.1**	13.2	13.2	13.2	13.2
E	6.0	6.0	6.0	6.0	6.0	11.0	11.0	11.0	11.0	11.0
F	0.9	0.9	0.9	0.9	0.9	**4.5**	4.4	4.1	7.2	7.2
G						**4.4**	3.5	-	3.5	4.4
H										3.8
**Total**	**130.5 kb**	**129.6 kb**	**130.6 kb**	**131.2 kb**	**130.7 kb**	**130.3 kb**	**129.5 kb**	**130.5 kb**	**131.1 kb**	**130.6**

* The differences in the genome of HearNPV-TR were remarked as bold in the table.

## Discussion

A thorough understanding of the biological and molecular properties of a baculovirus is a vital prerequisite to the development of an effective biopesticide against economically important insect pests and is crucial to designing intelligent strategies in pest management. Knowing the genome of the microorganism to be used for biopesticide production provides many advantages. For example, knowing the host spectrum and the number and copy of virulence-enhancing genes can give an idea of the strength of the pesticide to be produced. In this study, *hr*1 and *hr*3 regions in the HearNPV-TR genome and *bro*-a gene are different from other Helicoverpa NPV genomes. This may cause virulence differences among viruses. Many baculoviruses of the *Helicoverpa* species have been developed as commercial biopesticides [34–37]. As with other baculoviruses, the isolates from *Helicoverpa* species appear to have different virulence characteristics against the natural host.

To date, complete genome sequences of twenty-five different isolates from Helicoverpa SNPVs have been reported. They belong to viruses isolated from *Helicoverpa armigera*, *H*. *zea* and *H*. *assulta* hosts. Three of these isolates are from China [38, 39], twelve from Australia [40–43], one from Kenya [44], one from India [45], one from Russia (www.ncbi.nlm.nih.gov/nuccore/KJ004000), one from USA [46] and one from Brazil [47]. Differences among the genomes of these isolates are mostly in the *bro* genes and *hrs* [38, 41–46].

HearNPV-TR showed high virulence and effective against all *Helicoverpa* species distributed in Turkey [12], which made it an excellent candidate as a pest control agent. In this study, the genomic properties of HearNPV-TR were expounded in detail and compared to the genomes of four Helicoverpa SNPVs (HasNPV, HaSNPV-C1, HaSNPV-G4, HaSNPV-AU).

The genome of HearNPV-TR has 126, 132, 134, and 134 homologous ORFs to HasNPV, HaSNPV-C1, HaSNPV-G4 and HaSNPV-AU, respectively (S4 Table). Additionally, nucleotide sequence identities with the above isolates are 98%, 98%, 99% and 99%, respectively. Kimura analysis also supported the similarity of HearNPV-TR to the HearNPV isolates reported in the literature. However, HearNPV-TR genome revealed differences in *hr3*, *hr5* regions and in *bro*-a gene (S1 Table).

Regions of *hrs* have high A+T contents and have been implicated as transcriptional enhancers [23, 48, 49]. Additionally, these regions affect virulence and progeny virus production [50]. The number of *hrs* was determined to be between 2 and 17 in all sequenced baculovirus genomes [51]. *hrs* were not found in Chrysodeixis chalcites NPV (ChchNPV), Clanis bilineata NPV (ClbiNPV), Trichoplusia ni NPV (TniSNPV), Adoxophyes orana GV (AdorGV), Cydia pomonella GV (CpGV) and Spodoptera litura GV (SpltGV) genomes [52–55]. The HearNPV-TR genome contains 5 *hrs* (*hr1*-*hr5*). *Hr3* and *hr5* were different from other HearNPV. They also exhibited 87–89% and 77–89% similarities to those of other HearNPV genomes. Also, the size of all *hrs*, varied significantly among these genomes.

*Bro* genes have been reported to encode DNA binding proteins and affect host DNA replication and transcription [56]. The number of *bro* genes in different baculoviruses varies from none to 16 copies in LdMNPV [57–61]. HearNPV genomes contain two *bro* genes. The *bro*-a gene in HearNPV-TR is smaller than those in the other HearNPVs. Homology of amino acid encoded by HearNPV-TR *bro*-a gene to homologues in the other genomes is 83–86%. Similarities of *bro*-b encoded amino acid is in the 99% range (S1 Table). The HearNPV-TR *bro*-a clustered together in the phylogenetic tree with Heliothis virescens ascovirus 3i isolate (HvAV-3i). Ascovirus identity is also detected at Urbanus proteus nucleopolyhedrovirus (UrprNPV) *bro*-a gene [62]. This suggests a horizontal gene transfer from ascoviruses to baculoviruses.

A phylogenetic tree was generated based on concatenated amino acid sequences encoded by the 38 core genes from 25 Helicoverpa NPV genomes isolated in 8 different countries. The Turkish isolate clustered close to the isolates from China (H. assulta NPV, H. armigera NPV-C1 and -G4) and Australia (H. armigera NPV-AU).

Conventionally, baculoviruses have been named after the host species from which they were first isolated. However, Kimura-2 parameter analysis showed that many isolates are actually variants rather than distinct species [29, 30, 63]. Consequently, based on Kimura-2 parameters, NPV isolates that have a nucleic acid transition ratio under a certain value are accepted as one species and should be given the same name [64]. The data reported here based on analysis of concatenated sequences of proteins encoded by the 38 core genes confirmed that HearNPV-TR is a variant of Helicoverpa NPV and not a distinct species.

Restriction endonuclease profiles of HearNPV-TR, HasNPV, HaSNPV-C1, -G4 and -AU genomes, showed some differences in the isolates particularly in the XhoI profile (Table 2).

As a result of comparison of all ORFs and homologous repeat regions, significant differences were observed between genomes especially in the *hr3*, *hr5* and *bro*-a regions. Accordingly, genomic varieties between different variants of the same baculovirus species are generally thought to result from changes in *hrs* regions and *bro* genes, which could have consequences on virulence.

In this study, a complete genome analysis of HearNPV-TR was performed. Its high virulence to *Helicoverpa* species triggered our interest to develop it as a biological control agent. Also, our study provides a basis to further investigate the functions of the hypothetical genes in the genome.

## Supporting information

S1 Table Baculovirus genomes used in phylogenetic analysis of HearNPV-TR. (PDF)

S2 Table Baculovirus genomes used in phylogenetic analysis of HearNPV-TR. (PDF)

S3 Table Kimura-2 parameter analysis of HearNPV-TR with other baculovirus genomes. (PDF)

S4 Table Open reading frames (ORFs) and properties of the HearNPV-TR genome. (PDF)
