# Supplementary material for: Genome sequence analysis of a Helicoverpa armigera single nucleopolyhedrovirus (HearNPV-TR) isolated from Heliothis peltigera in Turkey
Source: PLoS One. 2020 Jun 12;15(6):e0234635. doi: 10.1371/journal.pone.0234635 (PMC7292396; doi:10.1371/journal.pone.0234635)
Supplement: S1 Table — (PDF) [file pone.0234635.s001.pdf]

Supplementary Table 1. Diversity of *bro* genes and *hrs* regions in HearNPV-TR and other HearSNPV strains

Virus strains	*bro* genes	aa sizes	identity	*hrs* regions	bp sizes	identity
**HearNPV-TR**	*bro*-a<br>*bro*-b	142<br>501	-<br>-	*hr*1<br>*hr*2<br>*hr*3<br>*hr*4<br>*hr*5	2264<br>1149<br>714<br>3246<br>2424	-<br>-<br>-<br>-<br>-
**HaSNPV**	*bro*-a<br>*bro*-b	358<br>501	**86 %**<br>99 %	*hr*1<br>*hr*2<br>*hr*3	1960<br>906<br>1187	97 %<br>99 %<br>**87 %**
**HaSNPV-C1**	**bro*-a<br>*bro*-a<br>*bro*-b	245<br>358<br>501	-<br>**86 %**<br>99 %	*hr*1<br>*hr*2<br>*hr*3<br>*hr*4<br>*hr*5	1969<br>907<br>297<br>2253<br>899	97 %<br>99 %<br>**89 %**<br>97 %<br>**77 %**
**HaSNPV-G4**	**bro*-a<br>*bro*-a<br>*bro*-b	245<br>528<br>501	-<br>**86 %**<br>99 %	*hr*1<br>*hr*2<br>*hr*3<br>*hr*4<br>*hr*5	2282<br>1150<br>759<br>2304<br>2806	98 %<br>98 %<br>**89 %**<br>92 %<br>**89 %**
**HaSNPV-AU**	*bro*-a<br>*bro*-b	550<br>501	**86 %**<br>99 %	*hr*1<br>*hr*2<br>*hr*3<br>*hr*4<br>*hr*5	2321<br>1150<br>755<br>2598<br>2806	97 %<br>98 %<br>**89 %**<br>98 %<br>**89 %**
**HaSNPV-AC53**	*bro*-a<br>**bro*-a<br>*bro*-b	237<br>363<br>501	**84 %**<br>-<br>99 %	*hr*1<br>*hr*2<br>*hr*3<br>*hr*4<br>*hr*5	1926<br>2377<br>482<br>2177<br>1385	97 %<br>99 %<br>**86 %**<br>97 %<br>**75 %**
**HaSNPV-AC53-C1**	*bro*-a<br>**bro*-a<br>*bro*-b	235<br>375<br>501	**83 %**<br>-<br>99 %	*hr*1<br>*hr*2<br>*hr*3<br>*hr*4<br>*hr*5	1928<br>2385<br>480<br>2178<br>1391	97 %<br>99 %<br>**89 %**<br>97 %<br>**77 %**
**HaSNPV-AC53-C5**	*bro*-a<br>**bro*-a<br>*bro*-b	235<br>375<br>501	**83 %**<br>-<br>99 %	*hr*1<br>*hr*2<br>*hr*3<br>*hr*4<br>*hr*5	1928<br>2385<br>480<br>2178<br>1391	97 %<br>99 %<br>**89 %**<br>97 %<br>**77 %**
**HaSNPV-AC53-C6**	*bro*-a<br>**bro*-a<br>*bro*-b	235<br>375<br>501	**83 %**<br>-<br>99 %	*hr*1<br>*hr*2<br>*hr*3<br>*hr*4<br>*hr*5	1928<br>2386<br>480<br>2178<br>1390	97 %<br>99 %<br>**89 %**<br>97 %<br>**77 %**

**HaSNPV-AC53-T4.1**	*bro*-a<br>**bro*-a<br>*bro*-b	235<br>375<br>501	**83 %**<br>-<br>99 %	*hr*1<br>*hr*2<br>*hr*3<br>*hr*4<br>*hr*5	1928<br>2386<br>480<br>2178<br>1391	97 %<br>99 %<br>**89 %**<br>97 %<br>**77 %**
**HaSNPV-AC53-T5**	*bro*-a<br>**bro*-a<br>*bro*-b	235<br>375<br>501	**83 %**<br>-<br>99 %	*hr*1<br>*hr*2<br>*hr*3<br>*hr*4<br>*hr*5	1928<br>2385<br>480<br>2178<br>1391	97 %<br>99 %<br>**89 %**<br>97 %<br>**77 %**
**HaSNPV-AC53-C9**	*bro*-a<br>**bro*-a<br>*bro*-b	235<br>375<br>501	**83 %**<br>-<br>99 %	*hr*1<br>*hr*2<br>*hr*3<br>*hr*4<br>*hr*5	1928<br>2386<br>480<br>2178<br>1391	97 %<br>99 %<br>**89 %**<br>97 %<br>**77 %**
**HaSNPV-AC53-T2**	*bro*-a<br>**bro*-a<br>*bro*-b	235<br>375<br>501	**83 %**<br>-<br>99 %	*hr*1<br>*hr*2<br>*hr*3<br>*hr*4<br>*hr*5	1928<br>2384<br>480<br>2178<br>1391	97 %<br>99 %<br>**89 %**<br>97 %<br>**77 %**
**HaSNPV-AC53-T4.2**	*bro*-a<br>**bro*-a<br>*bro*-b	235<br>375<br>501	**83 %**<br>-<br>99 %	*hr*1<br>*hr*2<br>*hr*3<br>*hr*4<br>*hr*5	1928<br>2384<br>480<br>2178<br>1391	97 %<br>99 %<br>**89 %**<br>97 %<br>**77 %**
**HaSNPV-AC53-C3**	*bro*-a<br>**bro*-a<br>*bro*-b	235<br>375<br>501	**83 %**<br>-<br>99 %	*hr*1<br>*hr*2<br>*hr*3<br>*hr*4<br>*hr*5	1928<br>2386<br>480<br>2178<br>1391	97 %<br>99 %<br>**89 %**<br>97 %<br>**77 %**
**HaSNPV-H25EA1**	*bro*-a<br>**bro*-a<br>*bro*-b	237<br>363<br>501	**83 %**<br>-<br>99 %	*hr*1<br>*hr*2<br>*hr*3<br>*hr*4<br>*hr*5	1926<br>2377<br>482<br>2177<br>1385	97 %<br>99 %<br>**86 %**<br>97 %<br>**75 %**
**HaSNPV-LB1**	*bro*-a<br>**bro*-a<br>*bro*-b	361<br>352<br>501	**86 %**<br>-<br>99 %	*hr*1<br>*hr*2<br>*hr*3<br>*hr*4<br>*hr*5	1853<br>1189<br>296<br>1728<br>2083	97 %<br>99 %<br>**87 %**<br>97 %<br>**77 %**
**HaSNPV-SP1A**	*bro*-a<br>**bro*-b<br>**bro*-c<br>*bro*-d	357<br>101<br>352<br>501	**83 %**<br>-<br>-<br>99 %	*hr*1<br>*hr*2<br>*hr*3<br>*hr*4<br>*hr*5	2068<br>1193<br>296<br>1728<br>2083	97 %<br>99 %<br>**88 %**<br>97 %<br>**79 %**
**HaSNPV-SP1B**	*bro*-a<br>**bro*-b<br>**bro*-c	237<br>88<br>206	**83 %**<br>-<br>-	*hr*1<br>*hr*2<br>*hr*3	1885<br>1193<br>295	97 %<br>99 %<br>**88 %**

	bro-d	501	99 %	*hr*4<br>*hr*5	1728<br>2076	97 %<br>**79 %**
**HaSNPV-LB3**	*bro*-a<br>**bro*-b<br>*bro*-c	360<br>352<br>501	**86 %**<br>-<br>99 %	*hr*1<br>*hr*2<br>*hr*3<br>*hr*4<br>*hr*5	1749<br>780<br>295<br>1300<br>2106	97 %<br>99 %<br>**87 %**<br>97 %<br>**77 %**
**HaSNPV-LB6**	*bro*-a<br>**bro*-b<br>*bro*-c	360<br>352<br>501	**86 %**<br>-<br>99 %	*hr*1<br>*hr*2<br>*hr*3<br>*hr*4<br>*hr*5	1822<br>780<br>295<br>1300<br>2081	97 %<br>99 %<br>**87 %**<br>97 %<br>**77 %**
**HaSNPV-NNg1**	*bro*-a<br>**bro*-b<br>**bro*-c<br>*bro*-d	360<br>101<br>352<br>501	**83 %**<br>-<br>-<br>99 %	*hr*1<br>*hr*2<br>*hr*3<br>*hr*4<br>*hr*5	2001<br>1396<br>133<br>1734<br>1135	97 %<br>99 %<br>**83 %**<br>97 %<br>**75 %**
**HaSNPV-L1**	*bro*-a<br>**bro*-b<br>*bro*-c	361<br>348<br>501	**86 %**<br>-<br>99 %	*hr*1<br>*hr*2<br>*hr*3<br>*hr*4<br>*hr*5	1853<br>1189<br>296<br>1728<br>2083	97 %<br>99 %<br>**88 %**<br>97 %<br>**75 %**
**HaSNPV-HS18**	*bro*-a<br>**bro*-b<br>*bro*-c	211<br>352<br>501	**86 %**<br>-<br>99 %	*hr*1<br>*hr*2<br>*hr*3<br>*hr*4<br>*hr*5	1926<br>2378<br>482<br>2177<br>1385	97 %<br>99 %<br>**89 %**<br>97 %<br>**76 %**
**HzSNPV-F16**	*bro*-a<br>**bro*-b<br>*bro*-c	211<br>352<br>501	**84 %**<br>-<br>99 %	*hr*1<br>*hr*2<br>*hr*3<br>*hr*4<br>*hr*5	1926<br>2378<br>482<br>2177<br>1385	97 %<br>99 %<br>**84 %**<br>97 %<br>**76 %**
**HzSNPV-Br/South**	**bro*-a<br>*bro*-b<br>**bro*-c<br>*bro*-d	138<br>211<br>352<br>501	-<br>**83 %**<br>-<br>99 %	*hr*1<br>*hr*2<br>*hr*3<br>*hr*4<br>*hr*5	494<br>2378<br>483<br>2179<br>1387	91 %<br>99 %<br>**84 %**<br>97 %<br>**76 %**

The * symbol indicates the absent genes in HearNPV-TR genome

The high differences between genomes identity were remarked as bold in the table
