# Supplementary material for: Genome sequence analysis of a Helicoverpa armigera single nucleopolyhedrovirus (HearNPV-TR) isolated from Heliothis peltigera in Turkey
Source: PLoS One. 2020 Jun 12;15(6):e0234635. doi: 10.1371/journal.pone.0234635 (PMC7292396; doi:10.1371/journal.pone.0234635)
Supplement: S2 Table — (PDF) [file pone.0234635.s002.pdf]

Supplementary Table 1. Baculovirus genomes used in phylogenetic analysis of HearNPV-TR

Nomenclature	Isolate name	Genome size	G/C content (%)	Nucleotide Identity (%)	Origin	Accesion number
**Alpha/I Baculoviruses**						
Autographa californica multiple nucleopolyhedrovirus	AcMNPV-C6	133.894	40.7	81	USA	L22858
Bombyx mori nucleopolyhedrovirus	BmNPV	126.879	40.3	76	India	JQ991010
Bombyx mandarina nucleopolyhedrovirus	BomaNPV	126.770	40.2	76	China	FJ882854
Choristoneura fumiferana multiple nucleopolyhedrovirus	CfMNPV	129.593	50.1	82	Canada	AF512031
Choristoneura murinana nucleopolyhedrovirus	ChmuNPV	124.688	50	70	Germany	KF894742
Hyphantria cunea nucleopolyhedrovirus	HycuNPV	132.959	45.5	75	Japan	AP009046
Maruca vitrata nucleopolyhedrovirus	MaviNPV	111.953	38.6	79	Taiwan	EF125867
**Alpha/II Baculoviruses**						
Agrotis ipsilon nucleopolyhedrovirus	AgipNPV-Illinois	155.122	48.6	81	USA	EU839994
Agrotis segetum nucleopolyhedrovirus	AgseNPV-A	147.544	45.7	72	Poland	DQ123841
Adoxophyes honmai nucleopolyhedrovirus	AdhoNPV	113.220	35.6	76	Japan	AP006270
Adoxophyes orana nucleopolyhedrovirus	AdorNPV	111.724	35	75	England	EU591746
Lymantria dispar multiple	LdMNPV-	162.658	57.3	79	Japan	KT626571

nucleopolyhedrovirus	3041					
Lymantria xylina multiple nucleopolyhedrovirus	LyxyMNPV-5	156.344	53.5	79	Taiwan	GQ202541
Mamestra brassicae multiple nucleopolyhedrovirus	MbMNPV-CHb1	154.451	40.1	69	China	JX138237
Mamestra configurata nucleopolyhedrovirus	MacoNPV-A 90/4	153.656	41.7	79	Canada	AF539999
Malacosoma neustria multiple nucleopolyhedrovirus	ManeNPV-T2	130.202	38.2	71	Turkey	KY968317
Pseudoplusia includens single nucleopolyhedrovirus	PsinSNPV-IE	139.132	39.3	78	Canada	KJ631622
Spodoptera exigua nucleopolyhedrovirus	SeMNPV HT-SeSP2A	135.395	43.9	77	Spain	HG425349
Spodoptera frugiperda multiple nucleopolyhedrovirus	SfMNPV	134.239	40.3	92	Colombian	KF891883
Spodoptera littoralis nucleopolyhedrovirus	SpliNPV-AN1956	137.998	44.7	89	Egpty	JX454574
Spodoptera litura nucleopolyhedrovirus	SpltNPV-G2	139.342	42.8	89	China	AF325155
Trichoplusia ni single nucleopolyhedrovirus	TnSNPV	134.394	39	69	USA	DQ017380
**Helicoverpa armigera nucleopolyhedrovirus** (include Helicoverpa zea single nucleopolyhedrovirus and Helicoverpa assulta single nucleopolyhedrovirus)	HearNPV-TR	130.691	38.9	*	Turkey	MK507817
	HaSNPV-AC53 (Vivus Max)	130.442	39.1	99	Australia	KJ909666
	HaSNPV-AC53-C1	130.460	39.1	99	Australia	KU738896
	HaSNPV-AC53-C5	130.442	39.1	99	Australia	KU738898
	HaSNPV-	130.435	39.1	99	Australia	KU738899

	AC53-C6					
	HaSNPV-AC53-T4.1	130.440	39.1	99	Australia	KU738902
	HaSNPV-AC53-T5	130.439	39.1	99	Australia	KU738904
	HaSNPV-AC53-C9	130.437	39.1	99	Australia	KU738900
	HaSNPV-AC53-T2	130.440	39.1	99	Australia	KU738901
	HaSNPV-AC53-T4.2	130.443	39.1	99	Australia	KU738903
	HaSNPV-AC53-C3	130.437	39.1	99	Australia	KU738897
	HaSNPV-H25EA1	130.436	39.1	99	Australia	KJ922128
	HaSNPV-AU	130.992	39	99	Australia	JN584482
	HaSNPV-LB1	131.966	39.2	99	Iberia	KJ701029
	HaSNPV-SP1A	132.481	39.2	99	Iberia	KJ701032
	HaSNPV-SP1B	132.265	39.2	99	Iberia	KJ701033
	HaSNPV-LB3	130.949	39.2	99	Iberia	KJ701030
	HaSNPV-LB6	130.992	39.2	99	Iberia	KJ701031
	HaSNPV-NNg1	132.425	39.2	99	Kenya	AP010907
	HaSNPV-C1	130.759	38.9	99	China	AF303045
	HaSNPV-G4	131.405	39	99	China	AF271059
	HaSNPV-L1	136.760	39.1	98	India	KT013224
	HzSNPV-HS18	130.890	39.1	99	Russia	KJ004000
	HzSNPV-F16 (Elcar/Biotrol)	130.869	39.1	99	USA	AF334030
	HzSNPV-	129.694	39.1	99	Brazil	KM596835

	Br/South					
	HasNPV-DJ0031	129.801	38.8	99	China	MG569706
	HearMNPV	154.196	40.1	68	China	EU730893
**Betabaculoviruses**						
Helicoverpa armigera granulovirus	HearGV	169.794	40.8	75	USA	EU255577
Cydia pomonella granulovirus	CpGV-M1	123.500	45.3	67	Mexican	U53466
Xestia c-nigrum granulovirus	XecnGV	178.733	40.7	69	USA	AF162221
