# Supplementary material for: Genome sequence analysis of a Helicoverpa armigera single nucleopolyhedrovirus (HearNPV-TR) isolated from Heliothis peltigera in Turkey
Source: PLoS One. 2020 Jun 12;15(6):e0234635. doi: 10.1371/journal.pone.0234635 (PMC7292396; doi:10.1371/journal.pone.0234635)
Supplement: S4 Table — (PDF) [file pone.0234635.s004.pdf]

Supplementary Table 3. Open reading frames (ORFs) and properties of the HearNPV-TR genome

					No ORF (% aa identities)					
	HearNPV-TR MK507817				AcMNPV L22858	HasNPV MG569706	HaSNPV-C1 AF303045	HaSNPV-G4 AF271059	HaSNPV-AU JN584482	CpGV NC_002816
ORF	Name	Position	Size (nt)	Size (aa)						
1	*polh*	1→741	741	247	8 (86)	1 (99)	1 (99)	1 (99)	1 (99)	1 (57)
2	*p78/83*	738←1988	1251	417	9 (27)	2 (95)	2 (94)	2 (96)	2 (96)	51 (34)
3	*pk-1*	1937→2806	870	290	10 (39)	3 (100)	3 (99)	3 (99)	3 (100)	3 (37)
4	*hoar*	2929←4908	1980	660		4 (92)	4 (94)	4 (91)	4 (92)	
5	*hypothetical*	5042→5218	177	59				5 (95)	5 (95)	
6	*hypothetical*	5364→6245	882	294		5 (95)	6 (95)	6 (95)	6 (94)	
7	*hypothetical*	6455←6616	162	54			7 (90)	7 (100)	7 (96)	
8	*ie-0*	6604→7461	858	286	141 (33)	6 (98)	8 (98)	8 (98)	8 (98)	
9	*p49*	7478→8884	1407	469	142 (50)	7 (99)	9 (99)	9 (99)	9 (99)	15 (34)
10	*odv-e18*	8895→9140	246	82	143 (75)	8 (100)	10 (100)	10 (99)	10 (100)	14 (46)
11	*odv-ec27*	9155→10009	855	285	144 (50)	9 (99)	11 (99)	11 (100)	11 (99)	97 (29)
12	*ac145*	10002→10331	330	110	145 (48)	10 (100)	12 (100)	12 (100)	12 (100)	9 (42)
13	*ac146*	10358←10969	612	204	146 (29)	11 (97)	13 (97)	13 (98)	13 (98)	
14	*ie-1*	10972→12957	1986	662	147 (34)	12 (95)	14 (96)	14 (97)	14 (97)	7 (22)
15	*pif-5/odv-e56*	13011←14075	1065	355	148 (52)	13 (97)	15 (97)	15 (97)	15 (97)	18 (44)
16	*me53*	14236→15315	1080	360	139 (24)	14 (99)	16-17 (98)	16-17 (98)	16 (98)	
17	*hypothetical*	15318→15485	168	56		15 (95)	18 (95)	18 (95)	17 (95)	
18	*hypothetical*	15538←15819	282	94		16 (94)	19 (94)	19 (94)	18 (94)	
19	*pif-0/p74*	15825→17906	2082	694	138 (53)	17 (99)	20 (99)	20 (98)	19 (99)	60 (40)
20	*p10*	17959←18267	309	103			21 (98)	21 (98)	20 (98)	
21	*p26*	18305←19108	804	268	136 (35)	19 (99)	22 (99)	22 (99)	21 (99)	
22	*hypothetical*	19220→19423	204	68				23 (99)	22 (99)	
23	*lef-6*	19500←20063	564	188	28 (32)	20 (99)	24 (99)	24 (99)	23 (99)	
24	*DNA binding protein*	20077←21048	972	324	25 (32)	21 (100)	25 (100)	25 (100)	24 (100)	
25	*ac26*	21192→21668	477	159	26 (37)	22 (99)	26 (99)	26 (99)	25 (99)	
	hr1 repeat region	21669-23932	2264							
26	*hypothetical*	23838→23990	153	51						
27	*hypothetical*	23933←24700	768	256	34 (37)	23 (100)	27 (100)	27 (99)	26 (99)	
28	*ubiquitin*	24540→24791	252	84	35 (75)	24 (100)	28 (100)	28 (100)	27 (100)	54 (78)
29	*hypothetical*	24837→25361	525	175		25 (100)	29 (100)	29 (100)	28 (100)	
30	*lese-25 like protein*	25381→25953	573	191		26 (99)	30 (99)	30 (99)	29 (98)	
31	*39k/pp31*	26017←26952	936	312	36 (34)	27 (99)	31 (99)	31 (99)	30 (99)	
32	*lef-11*	26918←27370	453	151		28 (98)	32 (98)	32 (98)	31 (98)	58 (38)
33	*ac38-like protein*	27270←27986	717	239	38 (52)	29 (99)	33 (99)	33 (99)	32 (99)	69 (44)
34	*hypothetical*	28217→29293	1077	359		30 (98)	34 (98)	34 (98)	33 (97)	
35	*p47*	29367←30605	1239	413	40 (54)	31 (99)	35 (99)	35 (98)	34 (99)	68 (44)
36	*lef-12*	30678→31349	672	224	41 (31)	32 (97)	36 (97)	36 (97)	35 (97)	
37	*ac43*	31435→31677	243	81	43 (33)	33 (96)	37 (96)	37 (96)	36 (96)	
38	*lef-8*	31674←34379	2706	902	50 (62)	34 (99)	38 (99)	38 (99)	37 (99)	131(50)
39	*hypothetical*	34330→35010	681	227		35 (99)	39 (99)	39 (99)	38 (100)	
40	*hypothetical*	35007→35303	297	99		36 (90)	40 (90)	40 (90)	39 (94)	

41	*chitinase*	35311←37038	1728	576	126 (66)	37 (99)	41 (98)	41 (98)	40 (99)	10 (59)
42	*ac52*	37115←37660	546	182	52 (27)	38 (99)	42 (99)	42 (99)	41 (99)	
43	*ac53*	37758→38186	429	143	53 (43)	39 (99)	43 (99)	43 (100)	42 (100)	
44	*hypothetical*	38193←39329	1137	379		40 (99)	44 (100)	44 (100)	43 (99)	
45	*hypothetical*	39337←39576	240	80		41 (100)	45 (100)	45 (100)	44 (100)	
46	*lef-10*	39494→39739	246	82	53a (38)	42 (99)	46 (99)	46 (100)	45 (100)	
47	*vp1054*	39612→40664	1053	351	54 (41)	43 (99)	47 (99)	47 (99)	46 (98)	138 (29)
48	*hypothetical*	40784→40990	207	69		44 (100)	48 (100)	48 (100)	47 (100)	
49	*ac56*	40991→41185	195	65	56 (37)	45 (100)	49 (100)	49 (100)	48 (98)	
50	*ac57*	41471→41962	492	164	57 (42)	46 (100)	50 (99)	50 (99)	49 (99)	
51	*ac59*	42043←42525	483	161	59 (46)	47 (100)	51 (99)	51 (97)	50 (97)	
52	*chaB*	42537←42806	270	90	60 (42)	48 (100)	52 (99)	52 (99)	51 (99)	26 (24)
53	*fp*	43018←43728	711	237	61 (63)	49 (100)	53 (100)	53 (100)	52 (98)	118 (34)
54	*hypothetical*	43801→44028	228	76			54 (100)	54 (100)	53 (98)	
55	*lef-9*	44138→45697	1560	520	62 (64)	50 (100)	55 (100)	55 (99)	54 (99)	117 (55)
56	*cathepsin*	45781←46884	1104	368	127 (46)	51 (100)	56 (99)	56 (99)	55 (100)	11 (41)
57	*hypothetical*	46925←47530	606	202		52 (99)	57 (99)	57 (99)	56 (99)	
58	*gp37*	47583←48422	840	280	64 (60)	53 (99)	58 (99)	58 (99)	57 (99)	13 (42)
	hr2 repeat region	48423-49571	1149							
59	*bro-a*	49572→49997	426	142		54 (86)	60 (86)	60 (86)	58 (86)	
	hr3 repeat region	50087-50800	714							
60	*hypothetical*	51605←51811	207	69						
61	*he65*	51860→52588	729	243	105 (34)	55 (99)	61 (100)	61 (100)	59 (100)	
62	*iap-2*	52664←53416	753	251	71 (31)	56 (99)	62 (99)	62 (99)	60 (99)	94 (30)
63	*putative methyl transferase*	53464←54309	846	282	69 (42)	57 (99)	63 (99)	63 (99)	61 (99)	
64	*pif-6*	54257←54658	402	134	68 (47)	58 (100)	64 (100)	64 (100)	62 (99)	114 (34)
65	*lef-3*	54669→55817	1149	383	67 (25)	59 (99)	65 (99)	65 (99)	63 (99)	
66	*desmoplakin*	55924←58281	2358	786	66 (29)	60 (99)	66 (99)	66 (99)	64 (99)	
67	*dnapol*	58312→61374	3063	1021	65 (46)	61 (99)	67 (99)	67 (99)	65 (99)	111 (37)
68	*ac74*	61451←61924	474	158	74 (26)	62 (100)	68 (100)	68 (100)	66 (100)	
69	*hypothetical*	61975←62367	393			63 (100)	69 (100)	69 (100)	67 (100)	
70	*ac76*	62364←62621	258	86	76 (40)	64 (100)	70 (100)	70 (100)	68 (100)	107 (38)
71	*vlf-1*	62662←63900	1239	413	77 (74)	65 (100)	71 (99)	71 (100)	69 (99)	106 (33)
72	*ac78*	63913←64257	345	115	78 (42)	66 (100)	72 (100)	72 (100)	70 (100)	
73	*gp41*	64314←65282	969	323	80 (59)	67 (100)	73 (100)	73 (100)	71 (100)	104 (35)
74	*ac81*	65212←65976	765	255	81 (52)	68 (100)	74 (100)	74 (99)	72 (99)	103 (49)
75	*telokin-like protein*	65810←66487	678	226	82 (34)	69 (100)	75 (99)	75 (99)	73 (99)	
76	*pif-8/vp91/p95*	66417→68867	2451	817	83 (40)	70 (99)	76 (99)	76 (99)	74 (99)	101 (31)
77	*hypothetical*	68870←69046	177	59						
78	*cg30*	69012←69926	915	305	88 (27)	71 (99)	77 (99)	77 (99)	75 (99)	
79	*vp39*	69952←70833	882	294	89 (43)	72 (99)	78 (100)	78 (100)	76 (100)	96 (32)
80	*lef-4*	70790→72217	1428	476	90 (44)	73 (99)	79 (99)	79 (100)	77 (99)	95 (32)
81	*p33*	72270←73034	765	255	92 (53)	74 (100)	80 (100)	80 (100)	78 (100)	93 (40)
82	*p18*	72994→73524	531	177	93 (55)	75 (100)	81 (100)	81 (100)	79 (100)	92 (35)
83	*odv-e25*	73570→74262	693	231	94 (42)	76 (100)	82 (100)	82 (100)	80 (100)	91 (52)
84	*hypothetical*	74294←74776	483	161		77 (96)	83 (96)	83 (96)	81 (95)	

85	*helicase*	74795←78556	3762	1254	95 (42)	78 (99)	84 (99)	84 (99)	82 (99)	90 (35)
86	*pif-4*	78513→79034	522	174	96 (48)	79 (100)	85 (100)	85 (99)	83 (99)	89 (41)
87	*38k*	79093←80178	1086	362	98 (44)	80 (99)	86 (99)	86 (99)	84 (99)	88 (39)
88	*lef-5*	79954→80901	948	316	99 (43)	81 (100)	87 (100)	87 (100)	85 (100)	87 (38)
89	*p6.9*	80895←81203	309	103		82 (88)	88 (94)	88 (94)	86 (94)	
90	*p40*	81268←82377	1110	370	101 (40)	83 (99)	89 (99)	89 (99)	87 (99)	85 (26)
91	*hypothetical*	82423←82791	369	123		84 (99)	90 (99)	90 (99)	88 (99)	
92	*p45/p48*	82791←83924	1134	378	103 (50)	85 (99)	91 (99)	91 (99)	89 (99)	83 (37)
93	*vp80*	84020→85837	1818	606		86 (99)	92 (99)	92 (99)	90 (99)	
94	*pif-7*	85834→86010	177	59	110 (32)		93 (98)	93 (98)	91 (98)	
95	*odv-ec43*	86025→87110	1086	362	109 (52)	87 (99)	94 (99)	94 (99)	92 (99)	55 (33)
96	*pif-9/ac108*	87156→87440	285	95	108 (47)	88 (100)	95 (100)	95 (100)	93 (100)	
97	*odv-e66*	87507←89525	2019	673	46 (43)	89 (99)	96 (99)	96 (99)	94 (99)	37 (44)
98	*p13*	89546←90376	831	277		90 (99)	97 (99)	97 (99)	95 (99)	47 (51)
	hr4 repeat region	89300-92545	3246							
99	*pif-3*	93623→94222	600	200	115 (43)	91 (100)	98 (100)	98 (100)	96 (100)	35 (35)
100	*hypothetical*	94226→94582	357	119		92 (95)	99 (94)	99 (94)	97 (94)	
101	*hypothetical*	94678→96210	1533	511		93 (99)	100 (99)	100 (99)	98 (99)	
102	*ac106*	96289→97050	762	254	106 (47)	94 (99)	101 (99)	101 (99)	99 (98)	52 (39)
103	*hypothetical*	97067→97399	333	111		95 (97)	102 (97)	102 (97)	100 (97)	
104	*iap-3*	97456←98262	807	269	27 (34)	96 (97)	103 (97)	103 (97)	101 (97)	17 (38)
105	*hypothetical*	98259←98552	294	98			104 (100)	104 (100)	102 (100)	
106	*bro-b*	98518←100023	1506	502	2 (36)	97 (99)	105 (99)	105 (99)	103 (99)	
107	*sod*	100173→100652	480	160	31 (76)	98 (99)	106 (99)	106 (98)	104 (99)	59 (63)
108	*hypothetical*	100659→102032	1374	458		99 (97)	107 (97)	107 (97)	105 (97)	
109	*hypothetical*	102067←102645	579	193		100 (98)	108 (98)	108 (99)	106 (99)	
110	*hypothetical*	102766→103161	396	132		101 (98)	109 (98)	109 (98)	107 (98)	
111	*ac117*	103139→103447	309	103	117 (33)	102 (100)	110 (100)	110 (100)	108 (100)	
112	*pif-1*	103515→105101	1587	529	119 (47)	103 (99)	111 (99)	111 (99)	109 (99)	75 (33)
113	*hypothetical*	105098→105334	237	79			112 (97)	112 (97)	110 (97)	
114	*fgf*	105357←106262	906	302	32 (28)	104 (97)	113 (97)	113 (98)	111 (98)	123 (23)
115	*alk-exo*	106395←107675	1281	427	133 (41)	105 (98)	114 (97)	114 (97)	112 (97)	125 (36)
116	*hypothetical*	107695←108084	390	130		106 (98)	115 (98)	115 (97)	113 (97)	
	hr 5 repeat region	107012-109435	2424							
117	*hypothetical*	110513←111439	927	309		107 (98)	115a (97)	115a (98)	114 (98)	
118	*ac111*	111638→111853	216	72	111 (36)	108 (97)	116 (99)	116 (99)	115 (99)	
119	*lef-2*	111962←112675	714	238	6 (41)	109 (96)	117 (96)	117 (96)	116 (99)	41 (27)
120	*p24*	113036→113779	744	248	129 (32)	110 (99)	118 (99)	118 (99)	117 (99)	71 (36)
121	*gp16*	113837→114127	291	97		111 (98)	119 (98)	119 (98)	118 (98)	
122	*calyx/pep*	114179→115201	1023	341	131 (30)	112 (100)	120 (99)	120 (99)	119 (100)	
123	*hypothetical*	115253→115744	492	164		113 (99)	121 (99)	121 (99)	120 (97)	
124	*odv-c21*	115874→116464	591	197		114 (97)	122 (96)	122 (97)	121 (97)	
125	*38.7k protein*	116508←117671	1164	388	13 (30)	115 (95)	123 (95)	123 (95)	122 (94)	
126	*lef-1*	117673←118410	738	246	14 (38)	116 (98)	124 (98)	124 (98)	123 (97)	74 (40)
127	*hypothetical*	118385←118822	438	146		117 (92)	125 (92)	125 (89)	124 (92)	
128	*egt*	118967→120514	1548	516	15 (44)	118 (98)	126 (98)	126 (98)	125 (98)	141 (42)

129	*hypothetical*	120672→121292	621	207		119 (99)	127 (99)	127 (99)	126 (99)	
130	*hypothetical*	121243→122043	801	267		120 (99)	128 (98)	128 (98)	127 (98)	
131	*hypothetical*	122113←124947	2835	945		121 (97)	129 (97)	129 (97)	128 (97)	
132	*pkip-1*	125285→125794	510	170		122 (99)	130 (99)	130 (96)	129 (99)	
133	*arif-1*	125912←126709	798	266	21 (26)	123 (98)	131 (98)	131 (97)	130 (98)	
134	*pif-2*	126981→128132	1152	384	22 (61)	124 (98)	132 (99)	132 (99)	131 (98)	48 (48)
135	*f-protein*	128173←130206	2034	678		125 (99)	133 (99)	133 (99)	132 (99)	31 (27)
136	*hypothetical*	130348←130893	546	182		126 (100)	134 (100)	134 (99)	133 (100)	
137	*hypothetical*	131075→131662	588	196		127 (100)	135 (98)	135 (97)	134 (98)	
