# Supplementary material for: Genome sequence analysis of a Helicoverpa armigera single nucleopolyhedrovirus (HearNPV-TR) isolated from Heliothis peltigera in Turkey
Source: PLoS One. 2020 Jun 12;15(6):e0234635. doi: 10.1371/journal.pone.0234635 (PMC7292396; doi:10.1371/journal.pone.0234635)
Supplement: S3 Table — (PDF) [file pone.0234635.s003.pdf]

Supplementary Table 2. Kimura-2 parameter analysis of HearNPV-TR with other baculovirus genomes

		1	2	3	4	5	6	7	8	9	10	11	12	13	14	15	16	17	18	19	20	21	22	23	24	25	26	27	28	29	30	31	32	33	34	35	36	37	38	39	40	41	42	43	44	45	46	47	48	49	50	51
1	**HearNPV-TR**																																																			
2	HaSNPV-AC53	**0,009**																																																	
3	HaSNPV-AC53-C1	**0,009**	0,000																																															
4	HaSNPV-AC53-C5	**0,009**	0,000	0,000																																													
5	HaSNPV-AC53-C6	**0,009**	0,000	0,000	0,000																																											
6	HaSNPV-AC53-T4.1	**0,009**	0,000	0,000	0,000	0,000																																									
7	HaSNPV-AC53-T5	**0,009**	0,000	0,000	0,000	0,000	0,000																																							
8	HaSNPV-AC53-C9	**0,009**	0,000	0,000	0,000	0,000	0,000	0,000																																						
9	HaSNPV-AC53-T2	**0,009**	0,000	0,000	0,000	0,000	0,000	0,000	0,000																																					
10	HaSNPV-AC53-T4.2	**0,009**	0,000	0,000	0,000	0,000	0,000	0,000	0,000	0,000																																				
11	HaSNPV-AC53-C3	**0,009**	0,000	0,000	0,000	0,000	0,000	0,000	0,000	0,000	0,000																																			
12	HaSNPV-H25EA1	**0,009**	0,002	0,002	0,002	0,002	0,002	0,002	0,002	0,002	0,002	0,002																																		
13	HaSNPV-AU	**0,008**	0,005	0,005	0,005	0,005	0,005	0,005	0,005	0,005	0,005	0,005	0,006																																	
14	HaSNPV-LB1	**0,009**	0,005	0,005	0,005	0,005	0,005	0,005	0,005	0,005	0,005	0,005	0,005	0,007																																
15	HaSNPV-SP1A	**0,009**	0,005	0,005	0,005	0,005	0,005	0,005	0,005	0,005	0,005	0,005	0,005	0,007	0,003																															
16	HaSNPV-SP1B	**0,009**	0,006	0,006	0,006	0,006	0,006	0,006	0,006	0,006	0,006	0,006	0,005	0,007	0,004	0,003																														
17	HaSNPV-LB3	**0,009**	0,005	0,005	0,005	0,005	0,005	0,005	0,005	0,005	0,005	0,005	0,005	0,007	0,004	0,004	0,004																													
18	HaSNPV-LB6	**0,009**	0,005	0,005	0,005	0,005	0,005	0,005	0,005	0,005	0,005	0,005	0,005	0,007	0,004	0,003	0,004	0,001																												
19	HaSNPV-NNg1	**0,009**	0,005	0,005	0,005	0,005	0,005	0,005	0,005	0,005	0,005	0,005	0,005	0,007	0,004	0,004	0,004	0,004	0,004																											
20	HaSNPV-C1	**0,022**	0,022	0,022	0,022	0,022	0,022	0,022	0,022	0,022	0,022	0,022	0,022	0,017	0,022	0,022	0,022	0,022	0,022	0,021																										
21	HaSNPV-G4	**0,008**	0,007	0,007	0,007	0,007	0,007	0,007	0,007	0,007	0,007	0,007	0,007	0,003	0,007	0,008	0,008	0,007	0,008	0,007	0,016																									
22	HaSNPV-L1	**0,012**	0,010	0,010	0,010	0,010	0,010	0,010	0,010	0,010	0,010	0,010	0,010	0,011	0,009	0,010	0,010	0,010	0,010	0,010	0,026	0,011																								
23	HzSNPV-HS18	**0,016**	0,009	0,009	0,009	0,009	0,009	0,009	0,009	0,009	0,009	0,009	0,008	0,013	0,012	0,012	0,012	0,012	0,012	0,012	0,029	0,014	0,017																							
24	HzSNPV-F16	**0,010**	0,002	0,002	0,002	0,002	0,002	0,002	0,002	0,002	0,002	0,002	0,002	0,006	0,005	0,005	0,006	0,005	0,005	0,005	0,022	0,007	0,010	0,007																						
25	HzSNPV-Br/South	**0,009**	0,002	0,002	0,002	0,002	0,002	0,002	0,002	0,002	0,002	0,002	0,001	0,006	0,005	0,005	0,005	0,005	0,005	0,005	0,022	0,007	0,010	0,007	0,000																					
26	HasNPV-DJ0031	**0,025**	0,026	0,026	0,026	0,026	0,026	0,026	0,026	0,026	0,026	0,026	0,026	0,021	0,026	0,026	0,026	0,026	0,026	0,026	0,005	0,021	0,030	0,034	0,026	0,026																									
27	HearMNPV	**0,706**	0,706	0,706	0,706	0,706	0,706	0,706	0,706	0,706	0,706	0,706	0,706	0,706	0,706	0,706	0,706	0,706	0,706	0,706	0,700	0,701	0,710	0,714	0,706	0,710	0,706																								
28	PsinSNPV-IE	**0,544**	0,545	0,545	0,545	0,545	0,545	0,545	0,545	0,545	0,545	0,545	0,544	0,544	0,545	0,545	0,544	0,544	0,544	0,544	0,558	0,545	0,543	0,551	0,544	0,544	0,561	0.735																							
29	TnSNPV	**0,545**	0,545	0,545	0,545	0,545	0,545	0,545	0,545	0,545	0,545	0,545	0,544	0,545	0,545	0,544	0,545	0,545	0,544	0,544	0,557	0,545	0,544	0,550	0,544	0,544	0,560	0.729	0,143																						
30	AgipNPV	**0,563**	0,564	0,564	0,564	0,564	0,564	0,564	0,564	0,564	0,564	0,564	0,564	0,564	0,563	0,563	0,563	0,562	0,562	0,563	0,579	0,564	0,563	0,571	0,564	0,564	0,583	0.635	0,479	0,480																					
31	AgseNPV-A	**0,560**	0,559	0,559	0,559	0,559	0,559	0,559	0,559	0,559	0,559	0,559	0,559	0,560	0,560	0,560	0,559	0,560	0,560	0,560	0,573	0,560	0,559	0,568	0,560	0,560	0,576	0.634	0,481	0,484	0,245																				
32	AdhoNPV	**0,534**	0,534	0,534	0,534	0,534	0,534	0,534	0,534	0,534	0,534	0,534	0,533	0,534	0,536	0,535	0,535	0,534	0,534	0,535	0,544	0,535	0,534	0,540	0,534	0,534	0,547	0.813	0,581	0,580	0,623	0,622																			
33	AdorNPV	**0,531**	0,532	0,532	0,532	0,532	0,532	0,532	0,532	0,532	0,532	0,532	0,531	0,532	0,533	0,532	0,532	0,532	0,531	0,533	0,542	0,532	0,532	0,538	0,532	0,532	0,545	0.810	0,583	0,581	0,624	0,620	0,056																		
34	LdMNPV-3041	**0,644**	0,642	0,642	0,642	0,642	0,642	0,642	0,642	0,642	0,642	0,642	0,642	0,643	0,641	0,642	0,642	0,641	0,641	0,641	0,657	0,643	0,643	0,651	0,643	0,643	0,662	0.880	0,623	0,628	0,525	0,550	0,696	0,705																	
35	LyxyMNPV-5	**0,619**	0,616	0,616	0,616	0,616	0,616	0,616	0,616	0,616	0,616	0,616	0,616	0,618	0,617	0,617	0,617	0,617	0,617	0,617	0,631	0,618	0,618	0,625	0,617	0,617	0,636	0.856	0,611	0,611	0,539	0,556	0,668	0,676	0,093																
36	MbMNPV-CHb1	**0,545**	0,546	0,546	0,546	0,546	0,546	0,546	0,546	0,546	0,546	0,546	0,546	0,546	0,546	0,546	0,546	0,546	0,546	0,545	0,560	0,546	0,546	0,554	0,546	0,546	0,563	0.272	0,477	0,476	0,379	0,379	0,592	0,589	0,629	0,612															
37	MacoNPV-A 90/4	**0,543**	0,543	0,543	0,543	0,543	0,543	0,543	0,543	0,543	0,543	0,543	0,543	0,543	0,544	0,544	0,543	0,542	0,543	0,542	0,558	0,544	0,543	0,552	0,544	0,543	0,560	0.362	0,479	0,478	0,364	0,371	0,595	0,594	0,603	0,592	0,097														
38	ManeNPV-T2	**0,542**	0,541	0,541	0,541	0,541	0,541	0,541	0,541	0,541	0,541	0,541	0,542	0,541	0,542	0,541	0,541	0,541	0,541	0,540	0,554	0,542	0,541	0,548	0,542	0,542	0,557	0.790	0,541	0,540	0,556	0,556	0,580	0,576	0,612	0,596	0,550	0,550													
39	SeMNPV HT-SeSP2A	**0,547**	0,546	0,546	0,546	0,546	0,546	0,546	0,546	0,546	0,546	0,546	0,546	0,547	0,547	0,546	0,546	0,545	0,545	0,546	0,562	0,547	0,546	0,554	0,546	0,546	0,566	0.650	0,472	0,472	0,302	0,314	0,608	0,608	0,568	0,566	0,397	0,389	0,541												
40	SfMNPV	**0,542**	0,542	0,542	0,542	0,542	0,542	0,542	0,542	0,542	0,542	0,542	0,542	0,543	0,543	0,543	0,542	0,542	0,542	0,543	0,557	0,542	0,544	0,550	0,542	0,542	0,560	0.657	0,479	0,478	0,335	0,341	0,601	0,599	0,607	0,598	0,406	0,402	0,541	0,271											
41	SpliNPV-AN1956	**0,591**	0,589	0,589	0,589	0,589	0,589	0,589	0,589	0,589	0,589	0,589	0,589	0,590	0,590	0,589	0,588	0,589	0,589	0,589	0,604	0,590	0,590	0,597	0,589	0,589	0,608	0.866	0,644	0,639	0,608	0,618	0,691	0,690	0,651	0,655	0,647	0,634	0,631	0,601	0,618										
42	SpltNPV-G2	**0,586**	0,585	0,585	0,584	0,585	0,585	0,585	0,585	0,585	0,585	0,584	0,585	0,585	0,585	0,584	0,584	0,585	0,585	0,585	0,599	0,585	0,586	0,593	0,585	0,584	0,602	0.861	0,646	0,639	0,615	0,624	0,683	0,681	0,664	0,663	0,638	0,628	0,629	0,609	0,616	0,110									
43	AcMNPV-C6	**0,639**	0,638	0,638	0,638	0,638	0,638	0,638	0,638	0,638	0,638	0,638	0,637	0,638	0,638	0,636	0,638	0,638	0,638	0,637	0,650	0,638	0,637	0,643	0,638	0,638	0,653	0.885	0,655	0,650	0,642	0,632	0,669	0,667	0,638	0,619	0,649	0,647	0,638	0,633	0,640	0,702	0,707								
44	BmNPV	**0,633**	0,632	0,632	0,632	0,632	0,632	0,632	0,632	0,632	0,632	0,632	0,631	0,632	0,632	0,630	0,632	0,632	0,632	0,631	0,643	0,632	0,631	0,637	0,632	0,632	0,647	0.886	0,654	0,649	0,644	0,633	0,663	0,661	0,643	0,621	0,651	0,648	0,633	0,637	0,641	0,703	0,703	0,040							
45	BomaNPV	**0,634**	0,633	0,633	0,633	0,633	0,633	0,633	0,633	0,633	0,633	0,633	0,632	0,633	0,633	0,631	0,633	0,633	0,633	0,632	0,644	0,633	0,632	0,638	0,633	0,633	0,648	0.888	0,653	0,649	0,647	0,633	0,665	0,663	0,644	0,621	0,652	0,648	0,634	0,638	0,641	0,703	0,704	0,039	0,008						
46	CfMNPV	**0,698**	0,698	0,698	0,698	0,698	0,698	0,698	0,698	0,698	0,698	0,698	0,699	0,699	0,698	0,698	0,698	0,698	0,698	0,697	0,710	0,697	0,698	0,704	0,699	0,699	0,713	0.944	0,717	0,717	0,642	0,653	0,744	0,744	0,599	0,600	0,704	0,690	0,701	0,666	0,686	0,743	0,751	0,422	0,432	0,433					
47	ChmuNPV	**0,698**	0,698	0,698	0,698	0,698	0,698	0,698	0,698	0,698	0,698	0,698	0,698	0,698	0,697	0,697	0,698	0,697	0,697	0,697	0,710	0,698	0,698	0,704	0,699	0,699	0,714	0.943	0,718	0,712	0,637	0,648	0,739	0,741	0,596	0,598	0,700	0,689	0,695	0,663	0,685	0,741	0,749	0,417	0,427	0,427	0,089				
48	HycuNPV	**0,684**	0,681	0,681	0,681	0,681	0,681	0,681	0,681	0,681	0,681	0,681	0,681	0,681	0,680	0,679	0,680	0,681	0,681	0,680	0,693	0,681	0,681	0,687	0,682	0,682	0,697	0.926	0,704	0,696	0,667	0,666	0,719	0,718	0,635	0,625	0,691	0,685	0,677	0,672	0,676	0,745	0,749	0,407	0,413	0,414	0,234	0,224			
49	MaviNPV	**0,632**	0,634	0,634	0,634	0,634	0,634	0,634	0,634	0,634	0,634	0,634	0,633	0,635	0,634	0,633	0,633	0,634	0,634	0,633	0,646	0,634	0,632	0,640	0,634	0,634	0,649	0.886	0,655	0,648	0,668	0,652	0,662	0,656	0,672	0,645	0,649	0,650	0,643	0,648	0,646	0,715	0,710	0,111	0,127	0,126	0,445	0,442	0,425		
50	HearGV	**0,859**	0,859	0,859	0,859	0,859	0,859	0,859	0,859	0,859	0,859	0,859	0,859	0,858	0,861	0,860	0,858	0,859	0,860	0,860	0,868	0,857	0,862	0,867	0,859	0,859	0,872	1.110	0,889	0,890	0,893	0,896	0,882	0,880	0,952	0,937	0,888	0,891	0,890	0,886	0,879	0,915	0,912	0,913	0,911	0,912	0,956	0,959	0,937	0,912		
51	CpGV-M1	**0,888**	0,889	0,889	0,889	0,889	0,889	0,889	0,889	0,889	0,889	0,889	0,888	0,887	0,888	0,888	0,889	0,887	0,887	0,888	0,898	0,888	0,888	0,897	0,889	0,889	0,901	1.137	0,923	0,923	0,872	0,890	0,932	0,929	0,902	0,900	0,921	0,906	0,911	0,892	0,899	0,907	0,923	0,935	0,937	0,935	0,953	0,949	0,963	0,949	0,612	
52	XecnGV	**0,854**	0,856	0,856	0,856	0,856	0,856	0,856	0,856	0,856	0,856	0,856	0,856	0,854	0,857	0,856	0,855	0,856	0,856	0,856	0,864	0,854	0,858	0,865	0,857	0,857	0,868	1.106	0,890	0,887	0,897	0,898	0,883	0,880	0,950	0,933	0,886	0,890	0,889	0,885	0,883	0,915	0,912	0,912	0,907	0,909	0,956	0,957	0,936	0,911	0,029	0,610
